# Reliability and state-dependency of EEG connectivity, complexity and network characteristics

**DOI:** 10.1038/s41598-025-23662-z

**Published:** 2025-11-04

**Authors:** L. S. Dominicus, D. Y. Lodema, B. Oranje, M. G. Zandstra, A. P. C. Hermans, L. Imhof, W. M. Otte, A. Hillebrand, K. Ambrosen, C. J. Stam, B. H. Ebdrup, E. van Dellen

**Affiliations:** 1 Department of Psychiatry, University Medical Center Utrecht, Utrecht, The Netherlands; 2 Center for Neuropsychiatric Schizophrenia Research (CNSR), and Center for Clinical Intervention and Neuropsychiatric Schizophrenia Research (CINS), Mental Health Center, Glostrup, Copenhagen University Hospital – Mental Health Services CPH, Copenhagen, Denmark; 3 Department of Child and Adolescent Psychiatry/Psychology, Erasmus University Medical Centre, Rotterdam, The Netherlands; 4 Department of Child Neurology, UMC Utrecht Brain Center, University Medical Center Utrecht, and Utrecht University, Utrecht, the Netherlands; 5 Amsterdam Neuroscience, Brain Imaging, Amsterdam, The Netherlands; 6 Amsterdam Neuroscience, Systems and Network Neurosciences, Amsterdam, The Netherlands; 7 Department of Clinical Neurophysiology and MEG Center, Department of Neurology, Amsterdam Neuroscience, Vrije Universiteit Amsterdam, Amsterdam UMC, Amsterdam, The Netherlands; 8 Department of Clinical Medicine, Faculty of Health and Medical Sciences, University of Copenhagen, Copenhagen, Denmark; 9 Department of Neurology, UZ Brussel and Vrije Universiteit Brussel, Brussels, Belgium

**Keywords:** Computational neuroscience, Biomedical engineering

## Abstract

Resting-state electroencephalography (EEG) metrics are influenced by task instructions and momentary factors such as cognitive state, complicating their use as biomarkers. We assessed how strongly three classes of resting-state EEG metrics depend on time and state: functional connectivity (FC; amplitude-envelope correlation, AECc, and phase-lag index, PLI), signal complexity (inverted joint permutation entropy, JPE_INV_, and permutation entropy, PE) and network topology derived from minimum spanning trees (MST). Sixty-four-channel EEG was recorded in healthy adults during two sessions six weeks apart (n = 42) and during semi-resting-state epochs embedded in a P50-gating task (n = 24). Reliability for repeated-resting recordings and resting-state versus semi-resting-state comparisons was quantified with intraclass correlation coefficients (ICC) at sensor and source level. PE showed consistently good-to-excellent reliability (ICC > 0.75–0.90). FC metrics ranged from poor to excellent, and MST metrics from poor to good. Across analyses, theta and alpha bands outperformed delta and beta bands. Alpha and theta PE and alpha PLI were the most robust, whereas MST and AECc require caution, especially outside theta and alpha bands. Our results identify theta and alpha PE and alpha PLI as robust measures suitable for biomarker development, while urging caution for MST and AECc due to their limited stability across time and state.

**Supplementary Information:**

The online version contains supplementary material available at 10.1038/s41598-025-23662-z.

## Introduction

Resting-state electroencephalography (EEG) measures intrinsic brain activity in the absence of explicit tasks orstimuli^1^. While resting-state EEG can be characterized by various aspects of brain function—from local oscillations to network-level organization^2^—its utility as a biomarker depends on measurement reliability. Subject heterogeneity, varying cognitive states and differences in measurement instructions can influence resting-state EEG recordings. These influences can lead to changes in amplitude, frequency distribution, oscillatory patterns and other EEG features^3,4^. This variability poses a challenge for biomarker development, where the goal is to reliably identify (alterations in) brain activity regardless of momentary state fluctuations. Resting-state EEG measures should demonstrate reliability across different conditions to serve as clinical biomarkers, indicating relative state independence while capturing the underlying trait^5,6^.

One way to assess EEG biomarker reliability is through measurement of their stability within subjects over time in repeated measures under similar conditions. Different EEG measures show varying degrees of temporal stability, which is high for power spectral characteristics, but variable for different functional connectivity (FC) measures^7^, where stability decreases with increasing retest intervals from hours to days^8–11^ to approximately 1 month^12,13^ or longer^10,14^. Among FC measures studied in EEG, phase-based measures such as phase lag index (PLI) show moderate to good reliability, while amplitude-based FC measures remain less thoroughly studied and show inconsistent reliability^10,13,15^. Complexity-based EEG measures have received limited attention, with one study reporting moderate reliability over a two-month period in a limited sample for several entropy measures^16^. Nevertheless, local signal complexity is an emerging type of EEG measure, possibly relating to balance between neuronal excitation and inhibition^17^. Expanding on this, an EEG marker combining local signal complexity and long-range FC is joint permutation entropy (JPE)^18^. Although JPE is a promising biomarker^19^, specifically inverted JPE (JPE_INV_), its temporal reliability remains unexplored in EEG.

Besides the functional interactions itself, the networks formed by interacting brain regions can also be characterized, although this often comes with arbitrary methodological choices^20^. Analyzing the core of such networks using Minimum Spanning Trees (MST) offers an alternative approach to characterize brain networks that avoids such choices^21^. This avoidance of arbitrary thresholds potentially leads to more consistent characterizations of core network topology across different studies, potentially reducing the heterogeneity observed in the literature on network alterations in brain disorders^22^. However, the temporal reliability of MST characteristics remains unclear. Nevertheless, three studies using a non-MST graph theory approach report heterogeneous outcomes ranging from poor to good reliability^9,14,23^.

The reliability of EEG measures may be improved through source reconstruction, which can reduce volume conduction effects while more directly capturing neuronal activity^3^, aid interpretation of results^24^ and increase signal-to-noise ratios^25^. Additionally, source reconstruction can lead to faster stabilization of MST measures^26^. Evidence showing reliability of measures based on source-reconstructed EEG is limited, with heterogeneous results ranging from poor to good^9,13,15^. Altogether, while power spectral measures show robust temporal reliability, evidence for the reliability of FC, complexity-based, and MST measures remains limited, with source reconstruction showing mixed benefits for measurement reliability.

Apart from these technical aspects, a complementary approach to assess biomarker stability is to investigate reliability of EEG measures through comparison of task-related resting periods, or semi-resting-state EEG^27^, with resting-state EEG. The periods surrounding task execution are characterized by heightened attention, anticipation, and cognitive preparation, potentially altering brain activity even in the absence of stimuli^28–31^. Evidence suggests that oscillatory neural fingerprints remain stable across resting-state EEG and semi-resting-state EEG in the context of a motor task^27^, indicating that resting-state EEG characteristics can maintain reliability despite different resting conditions. The extent to which more high-order measures such as FC, complexity and network characteristics remain stable across different resting conditions is yet to be investigated.

The present study used both repeated resting-state EEG measurements and semi-resting-state EEG to evaluate the reliability of various quantitative EEG measures across both time and different resting-state contexts in healthy subjects. The semi-resting-state data consisted of EEG in the periods of rest in a P50 gating paradigm measurement, which aims to measure sensory gating, referring to the preattentional filtering of irrelevant stimuli^32^. EEG offers diverse measures such as phase-, amplitude- and complexity-based measures, which fundamentally differ in the characteristics they capture^3^, alongside network topology methods like the widely applied Minimum Spanning Tree (MST) to characterize brain organization^22^. To cover these categories, we assessed phase-based (PLI), amplitude-based (corrected amplitude envelope correlation or AECc), and complexity-based (JPE_INV_) FC measures, alongside local signal complexity (PE) and network topology characteristics (MST), at both sensor and source level. This selection provides a diverse sample of common, relatively interpretable measures, aligning with prior applications in clinical contexts^19,33–35^. Given previous heterogeneous findings^7^, we hypothesized that (1) connectivity and complexity measures would demonstrate moderate reliability across both time and resting conditions and (2) MST measures would show comparable reliability as they are based on methodologically robust approach to network characterization.

## Methods

EEG recordings from healthy adults in the age range between 18 and 40 years were acquired as part of the Pan European Collaboration Antipsychotic-Naïve Studies (PECANS), ClinicalTrials.gov Identifier: NCT01154829)^36^ and the OPTIMISE STUDY (ClinicalTrials.gov Identifier: NCT01555814)^37,38^. Matching inclusion criteria and site allowed for the combination of these two datasets. Exclusion criteria included a presence or history of head injury or mental health conditions, physical illness and/or first-degree relatives with mental health conditions. Data were collected according to the declaration of Helsinki and both studies were approved by the Danish Ethical Committee. Participants signed informed consent before participating.

### EEG recordings

**Fig. 1:**
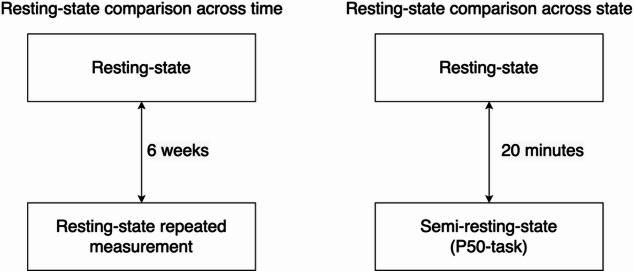
Overview of study design. Resting-state and repeated resting-state EEG measurements were made with a 6 week interval. Resting-state and semi-resting-state EEG measurements were made on the same day with an approximately 20 min interval.

EEG recordings were acquired using BioSemi hardware (BioSemi, Amsterdam, The Netherlands). A cap with 64active electrodes with an extended 10–20 layout was used. Sampling rate was 2048 Hz. Participants were seated in a comfortable chair in a sound-insulated room with a sound level of < 40 dB. Participants were instructed not to drink caffeine-containing beverages and/or smoke cigarettes 1 h before the recording. Participants were instructed to stay awake. EEG recordings took place between 9 AM and noon. An event-related potential (ERP) battery was recorded followed by ten minutes of eyes-closed resting-state EEG, ensuring a constant setting^39–43^. The ERP battery, also known as Copenhagen Psychophysiology Test Battery (CPTB), includes the startle reflex paradigm (PPI), P50 suppression, mismatch negativity (MMN), and selective attention paradigms. EEG recordings were made twice with a 6 week time interval, allowing for quantification of temporal reliability of the resting-state EEG characteristics described below. We analyzed epochs obtained during the rest periods of a P50 gating task in addition to true resting-state EEG. In this paper, we refer to the former as semi-resting-state EEG. We chose the P50 task for this semi-resting-state analysis, as there are periods of 10 s between sets of stimuli. Participants listened to three identical blocks of 40 auditory stimuli and were instructed to focus their gaze on a fixed point. These stimuli consisted of 1.5 ms-long bursts of white noise with an interstimulus interval of 500 ms. The analyses of the P50 paradigm have previously been published^39,40^; in the current project we made use of the EEG data recorded in the time between the trials. Using this paradigm in addition to resting-state EEG allowed us to study the effect of different brain states affected by measurement instruction and attention level. We compared these EEG epochs with the resting-state EEG recordings, both of which were acquired on the same day. In Fig. 1, an overview of the study design is provided.

### EEG preprocessing

The EEG data were preprocessed using functions from version 1.8.0 of the open-source *MNE* Python package^44^. This implementation is openly accessible via: https://github.com/yorbenlodema/EEG-Pype. In short, we manually selected bad channels for interpolation and selected 15 artifact-free 4-s-long epochs for further quantitative analysis. See supplements S1 for a full explanation of the approach used.

### EEG measures

Data were filtered in four frequency bands: delta (0.5–4 Hz), theta (4–8 Hz), alpha (8–13 Hz), and beta (13–20 Hz)^2,45^. We excluded frequency content above 20 Hz to reduce muscle activity^45^. EEG features were calculated using in-house developed software in Python, using functions from the *Scipy* package (available via https://github.com/yorbenlodema/EEG-Pype). The reliability of FC measures (PLI and AECc)^46–48^, MST characteristics based on these FC estimates using a maximum spanning tree approach^21,49^, and entropy or complexity-based measures (PE and JPE_INV_)^18,50^ were studied. As a supplementary analysis, the AECc was calculated both on separate epochs and one-minute concatenated epochs, which could result in higher reliability^15^. See supplement S2 for a full explanation of this analysis. MST was calculated with a maximum spanning tree approach (see supplement S1 for a full explanation). For the MST measures, we included both conventional quantitative measures and MST overlap. The MST overlap provides an additional way of quantifying MST reliability by looking at the overlap of the network backbone during different recordings and conditions and is similar to the approach by Govaarts et al.^51^. See Table 1 for an overview of the included EEG measures and their description. For more details concerning the EEG preprocessing and analysis see supplement S1.

**Table 1:** Overview of included EEG characteristics and their descriptions.

EEG characteristics	Description
Functional connectivity	The AECc was obtained by estimating the amplitude of the analytic signal for two signals, and subsequently computing the correlation between their envelopes. Normalized values range from 0 to 1. See for further AECc details Supplement S2.
	The PLI characterizes consistent phase differences between two signals, ignoring zero-lag correlations. Ranges vary from 0 (no synchronization, or perfect zero-lag correlation) to 1 (complete non-zero-lag phase locking).
	The PLI_MST_ and the AECc_MST_ represent the average of a functional connectivity metric that is calculated over the edges of the MST instead of using a whole-brain average.
Inverted joint permutation entropy (JPE_INV_)	JPE quantifies the shared complexity between two multivariate time series by analyzing their joint symbolic dynamics, providing a normalized measure of synchronization or independence between the systems. Inverting the JPE means higher JPE_INV_ values reflect higher functional connectivity.
Permutation entropy (PE)	PE quantifies the complexity of neural activity by analyzing the order of consecutive data points in a time series. It measures the unpredictability of distinct patterns formed by ranking these points, reflecting the level of local brain activity complexity. PE was included since it is necessary to optimally interpret JPE_INV_ results, and to provide a local measure of signal complexity.
*MST characteristics*	
Degree (k)	Measures the number of edges/links for each node divided by the maximum number of edges possible. The maximum degree (kmax), which is the highest degree in the MST, was used as a macroscale network characteristic.
Leaf fraction (LF)	The ratio of the number of leaf nodes (L) divided by the maximum possible leaf number (which is equal to the number of nodes − 1). A leaf node is a node with only one edge.
Diameter (D)	Refers to the largest distance between any two nodes in the MST backbone. It can be interpreted as a measure of efficiency, where a low diameter indicates an efficient information flow between brainregions.
Betweenness centrality (BC)	Fraction of all shortest paths that pass through a node. A leaf node has a BC of zero. The central node in a star-like network, is characterized by BC = 1. For the MST global measure, the highest BC (BCmax) is used.
Eccentricity (ECC)	The eccentricity of a node is defined as the length of the longest of the shortest paths from this node to any other node. Here, we used the mean ECC of all nodes.
Tree hierarchy (Th)	Defines the hierarchy of the MST organization as optimal topology. Th is calculated as Th = L/ (2 m BCmax), where m = number of edges.
MST overlap	To estimate not just MST-derived metric reliability, but also reliability of the global topology of the MST, MST overlap was analyzed. The MST overlap was computed by calculating the overlapping edges of individual MSTs, with the MSTs based on the average connectivity matrices for two recordings or conditions for each subject. Then, the mean overlap was analyzed across the different conditions.

Overview of all electroencephalography (EEG) measures analyzed in this study, including functional connectivity measures, complexity measures, and minimum spanning tree (MST) characteristics. For each measure, a brief description of its calculation method and interpretation is provided. *PLI* phase lag index, *AECc* corrected amplitude envelope correlation, *MST* minimum spanning tree.

### Quantification of reliability

In this paper, the term “reliability” refers to test–retest reliability. A method that is commonly used to assess reliability is the Intraclass Correlation Coefficient (ICC). This test was first introduced by Fisher^52^ and modern versions are often used for clinical purposes, including assessing the reliability of clinical measurements like EEG recordings^53,54^. Compared to Pearson’s correlation, ICC is less sensitive to bias^54^. We used a 2-way mixed-effects ICC model^54^. The level of reliability was based on the 95% confidence interval of the ICC: reliability scores lower than 0.50 were considered ‘poor’, scores from 0.50 to 0.75 ‘moderate’, scores from 0.76 to 0.90 ‘good’, and scores above 0.90 ‘excellent’^54^. ICC scores were calculated for all EEG measures except MST overlap. This comparison was made over time for the repeated resting-state EEG, using the resting-state and follow-up after six weeks. We also calculated the ICC scores across resting-state conditions, comparing resting-state and semi-resting-state data. To assess whether resting-state EEG is stable and relatively state-independent, we interpreted the four different possible combinations of high and low reliability together for the two comparisons. The following combinations are possible:

- High state reliability and high temporal reliability: possible biomarker.
- Low state reliability and high temporal reliability: possible biomarker, sensitive to resting-state instructions.
- High state reliability and low temporal reliability: possible cross-sectional marker, sensitive to time-variable factors.
- Low state reliability and low temporal reliability: poor reliability.

## Results

The reliability analysis over time included 42 participants, while 24 participants were included for the reliability analysis across resting-states. The mean age of participants was 24.2 years (SD 5.4) for the group that completed the repeated resting-state measurement and 26.0 years (SD 6.5) for the group that additionally completed the semi-resting-state condition. In the resting-state group 61.9% (N = 26) were male compared to 70.8% (N = 17) in the semi-resting-state group. See supplement S3 for 95% confidence intervals of all measures based on non-parametric bootstrapping with 1000 resamples. Note that these intervals vary across the different measures and tend to be wider for the across states comparison due to the smaller sample size.

### Phase lag index

At sensor level, the PLI showed moderate reliability in the theta and alpha bands across time. The PLI showed poor delta and beta band reliability. When compared between resting-state and semi-resting-state conditions, the PLI showed excellent reliability in the theta band, good reliability in the alpha band and moderate reliability in the delta and beta band.

**Fig. 2:**
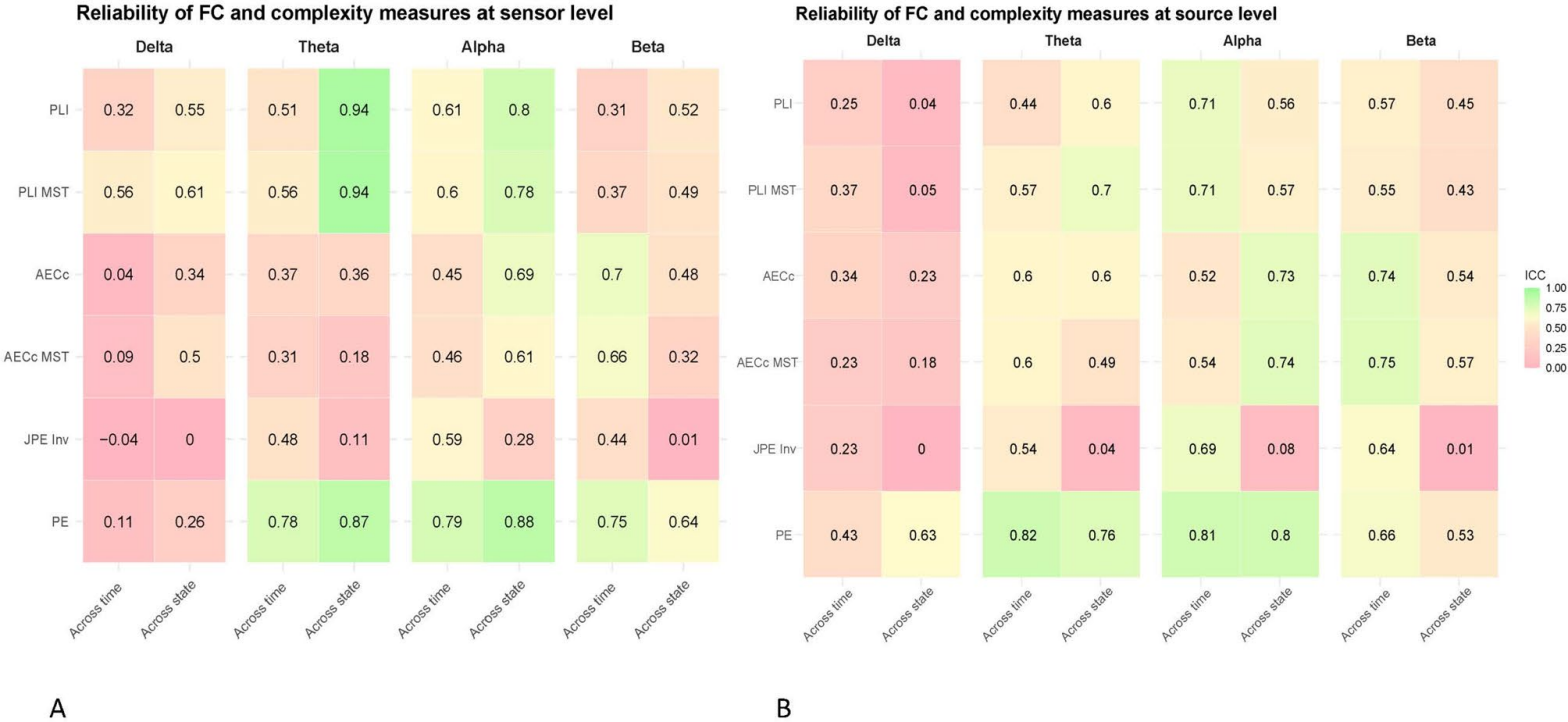
Temporal and state reliability of EEG measures at sensor and source level. Intraclass correlation coefficients (ICC) for EEG measures across time (6 week interval) and states (resting state vs. semi-resting-state) at both sensor level (**A**) and source level (**B**). Reliability scores lower than 0.50 were considered ‘poor’, scores from 0.50 to 0.75 as ‘moderate’, scores from 0.76 to 0.90 as ‘good’, and scores above 0.90 were considered ‘excellent’ (Koo & Li, 2016). Measures include phase lag index (PLI), corrected amplitude envelope correlation (AECc), permutation entropy (PE), and joint permutation entropy (JPE_INV_) across different frequency bands (delta: 0.5–4 Hz, theta: 4–8 Hz, alpha: 8–13 Hz, beta: 13–20 Hz). *MST* minimum spanning tree.

**Fig. 3:**
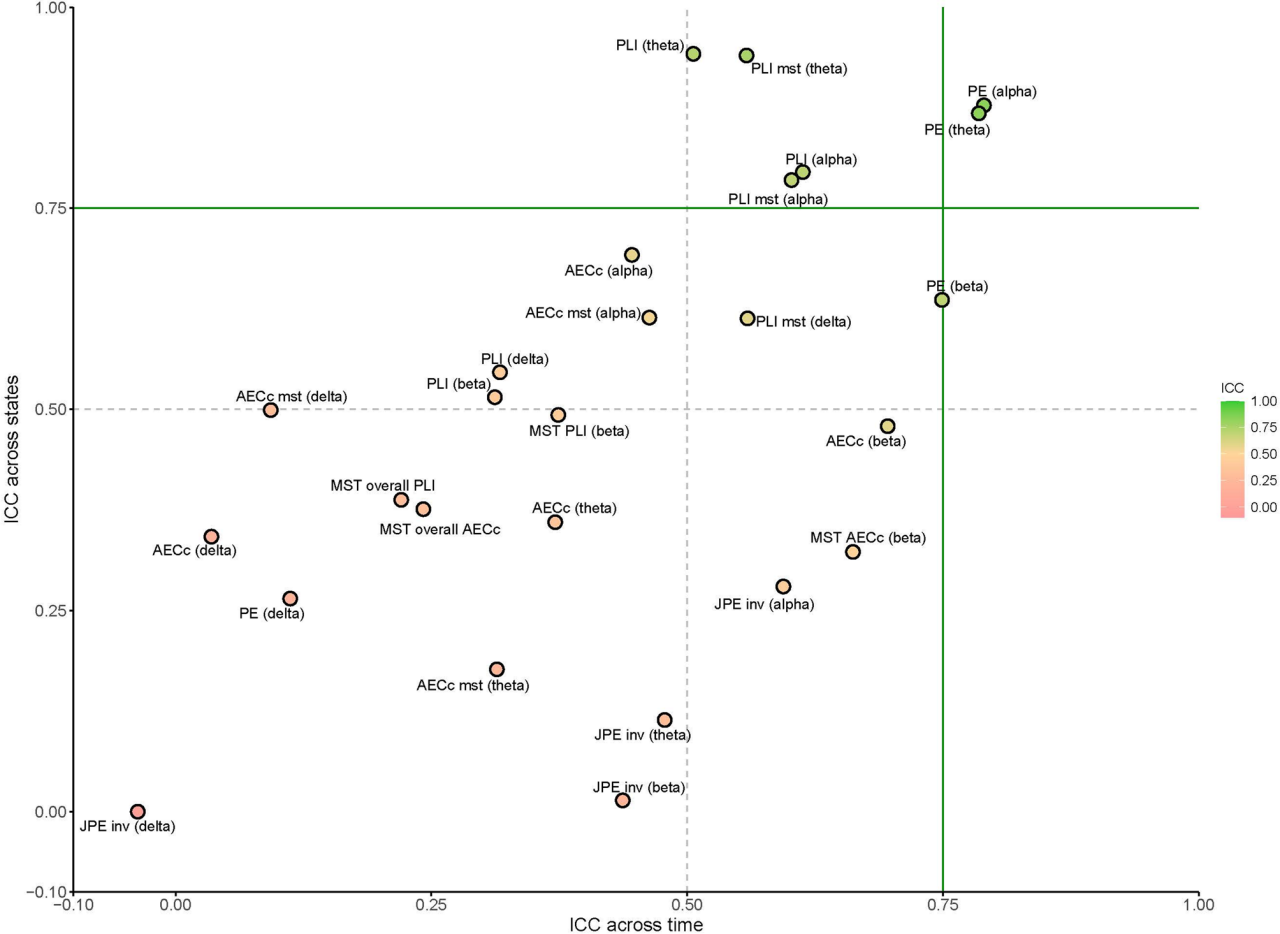
Scatterplot of measures across state and across time at sensor level. The ICCs for all MST measures, except for those for the PLI and AECc MST were averaged (‘overall’), as they all showed poor reliability. Delta: 0.5–4 Hz, theta: 4–8 Hz, alpha: 8–13 Hz, beta: 13–20 Hz). *PLI* phase lag index, *AECc* corrected amplitude envelope correlation, *MST* minimum spanning tree, *PE* permutation entropy, *JPE* joint permutation entropy, *ICC* intraclass correlation coefficients, *inv* inverted.

**Fig. 4:**
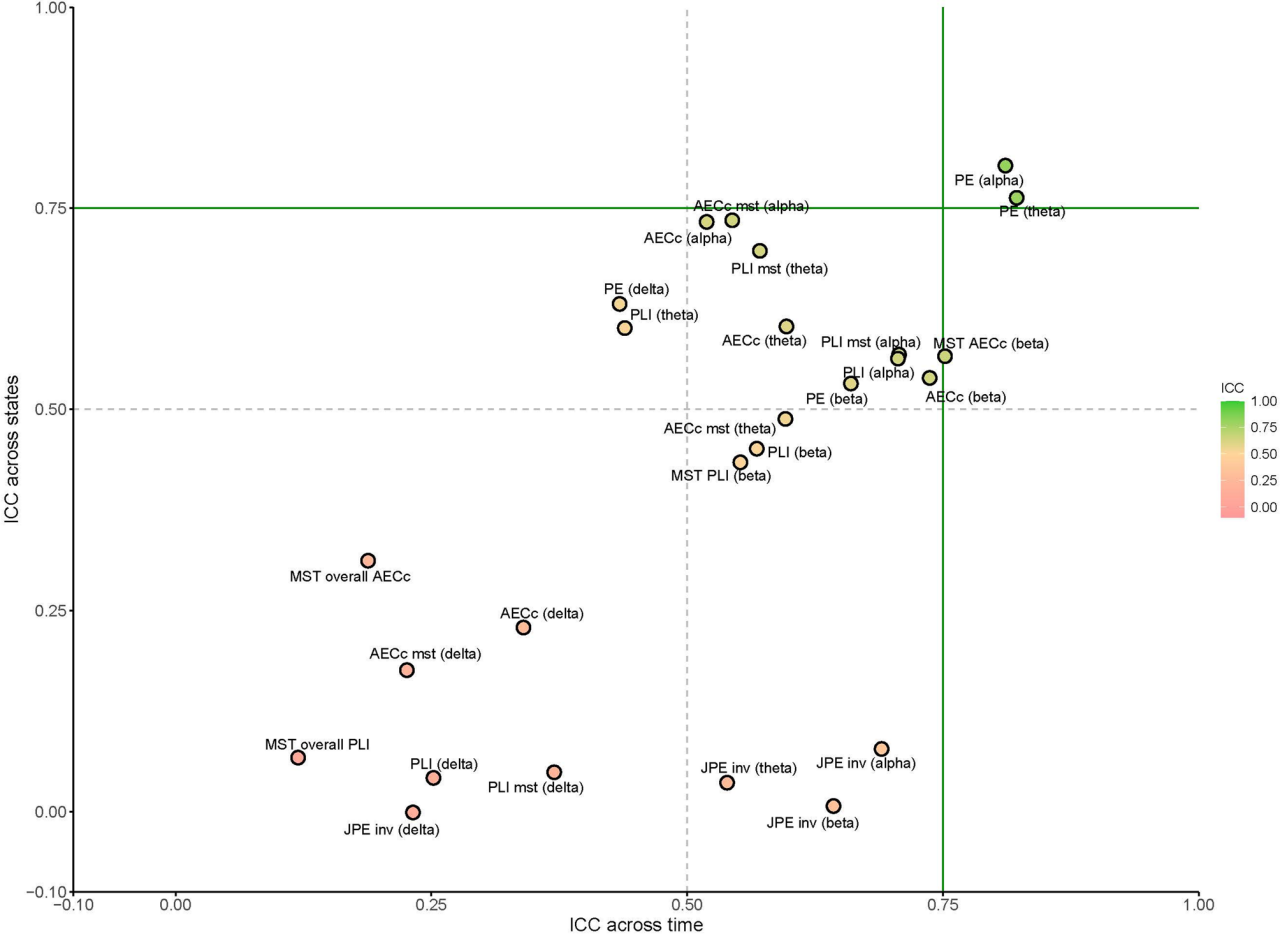
Scatterplot of measures across state and across time at source level. The ICCs for all MST measures, except for those for the PLI and AECc MST, were averaged (‘overall’), as they all, except for MST leaf fraction (based on alpha AECc), showed poor reliability. Delta: 0.5–4 Hz, theta: 4–8 Hz, alpha: 8–13 Hz, beta: 13–20 Hz). *PLI* phase lag index, *AECc* corrected amplitude envelope correlation, *MST* minimum spanning tree, *PE* permutation entropy, *JPE* joint permutation entropy, *ICC* intraclass correlation coefficients, *inv* inverted.

At source level, the PLI showed moderate reliability in the alpha and beta band when compared across time. In the delta and theta band the PLI showed poor reliability for this comparison. The PLI showed moderate to good reliability in the theta, alpha and beta bands and poor reliability in the delta band when compared across resting-states at source level. See Figs. 2, 3 and 4 for an overview of the results. 

**Fig. 5:**
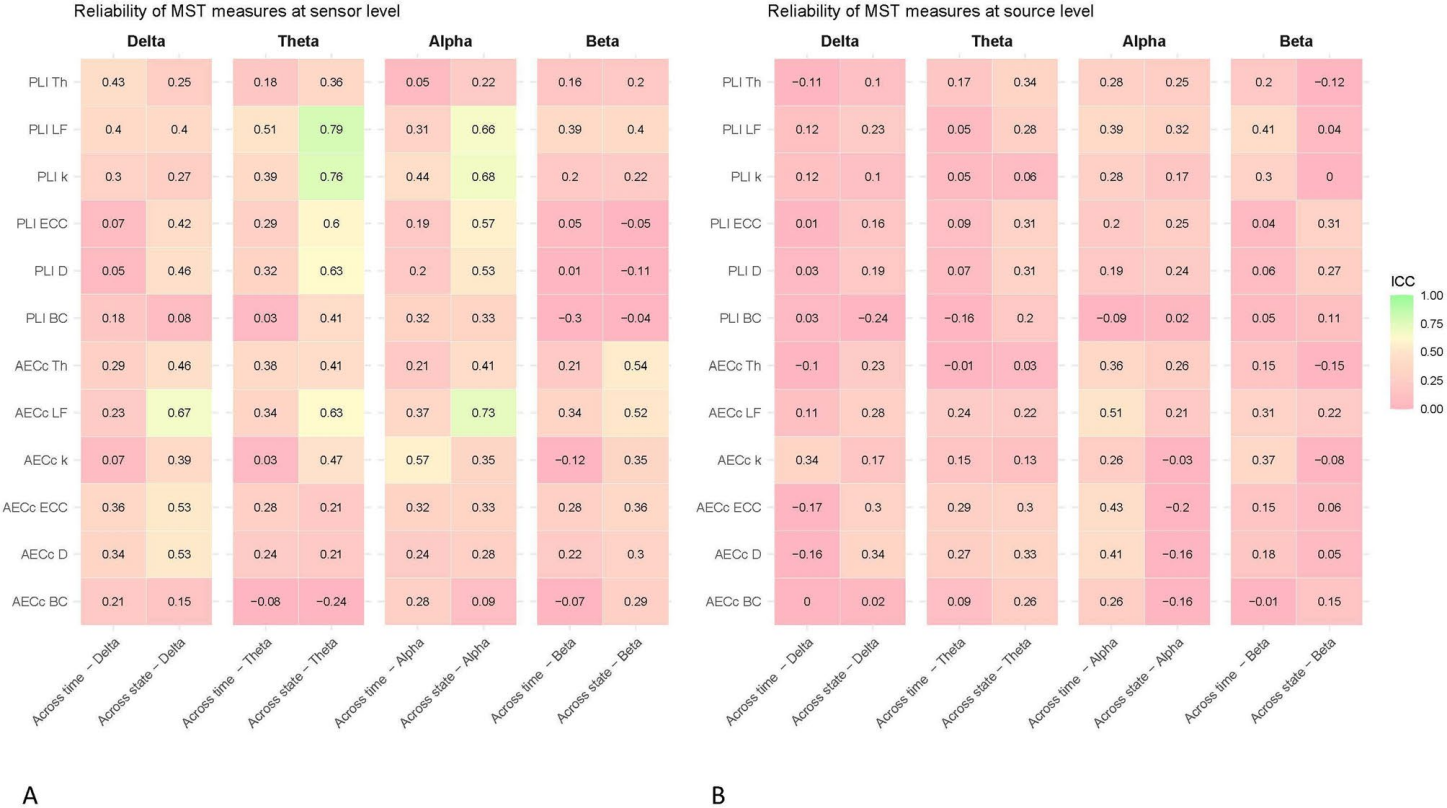
Temporal and state reliability of minimum spanning tree measures at sensor and source level. Intraclass correlation coefficients (ICC) for minimum spanning tree (MST) measures across time (6 week interval) and states (resting state vs. semi-resting-state) at both sensor level (**A**) and source level (**B**). Reliability scores lower than 0.50 were considered ‘poor’, scores from 0.50 to 0.75 as ‘moderate’, scores from 0.76 to 0.90 as ‘good’, and scores above 0.90 were considered ‘excellent’ (Koo & Li, 2016). MST measures were derived from both PLI and AECc connectivity matrices and include leaf fraction (LF), maximum degree (k), diameter (D), maximum betweenness centrality (BC), eccentricity (ECC), and tree hierarchy (Th) across different frequency bands (delta: 0.5–4 Hz, theta: 4–8 Hz, alpha: 8–13 Hz, beta: 13–20 Hz). *PLI* phase lag index, *AECc* corrected amplitude envelope correlation.

### Corrected amplitude envelope correlation

The AECc showed moderate reliability in the beta band compared across time at sensor level, while all other AECc frequency bands demonstrated poor reliability. Across-states comparisons at sensor level showed moderate AECc reliability in the alpha band, and poor reliability in the delta, theta, and beta bands.

At source level, the AECc demonstrated moderate reliability over time in the theta, alpha and beta band, and poor reliability in the delta band. The reliability across states was moderate-to-good in the alpha and beta band, but poor in the delta and theta band. See Figs. 2, 3 and 4 for an overview of the results. Concatenating epochs before calculating AECc yields mixed results and does not provide a substantial overall improvement. While concatenation may slightly increase reliability for state-level analyses in sensor space, it often performs comparably or significantly worse in other contexts, particularly at the source level. See Supplements S2 for full results.

### Permutation entropy and joint permutation entropy (inversed)

At sensor level, the PE showed good reliability over time in the theta and alpha bands, moderate in the beta band, and poor in the delta band. For JPE_INV_, reliability over time was moderate in the alpha band, but poor in the theta, beta, and delta bands. Across states, the reliability of PE was good in the theta and alpha bands, moderate in the beta band, and poor in the delta band. In contrast, JPE_INV_ showed poor reliability across states in all four frequency bands.

At source level, the PE demonstrated good reliability in the theta and alpha bands and moderate reliability in the beta band when compared across time; PE also showed poor reliability in the delta band across time. The JPE_INV_ showed moderate reliability when compared across time in the theta, alpha, and beta bands, and poor reliability in the delta band. When compared across resting-states at source level, the PE showed moderate reliability in the delta and beta bands and good reliability in the theta and alpha bands. The JPE_INV_ showed poor reliability across states for all frequency bands at the source level for this comparison. See Figs. 2, 3 and4 for an overview of the results.

### Minimum spanning tree

Several MST measures showed moderate reliability at sensor level. When compared across time, this included the AECc degree (k) in the alpha band and PLI Leaf fraction (LF) in the theta band. All other variables showed poor reliability for this comparison. When comparing across resting-states, some measures, mostly in the theta and alpha band, showed moderate to good reliability. This included the following measures: Leaf fraction (based on theta PLI, delta AECc, theta AECc, alpha PLI, alpha AECc, beta AECc), the maximum degree (based on theta PLI and alpha PLI), the diameter (based on delta AECc, theta PLI and alpha PLI) and eccentricity (based on delta AECc, theta PLI and alpha PLI) and tree hierarchy (based on beta AECc). See Figs. 3, 4 and 5 for a complete overview of these results.

At source level, the MST leaf fraction (based on alpha AECc) showed moderate reliability when compared across time. All other features showed poor reliability for this analysis.

### MST overlap

The MST overlap analysis generally showed low percentages of overlap between the MST matrix elements of AECc and PLI MST matrices for both comparisons. The overlap ranged from 2.7 to 10.8%, with the highest overlap in the alpha band, indicating that on average, no more than 6.8 edges were consistently present in this backbone network in repeated EEG registrations. Network reconstruction in source space led to less overlap between networks with a maximum overlap of 4.7% or 3.0 edges. See Table 2 for a complete overview of these results.

**Table 2:** Mean individual MST matrix overlap percentages across time and resting states.

	Overlap across repeated measurements (%) (N = 42)				Overlap across resting states (%) (N = 24)			
Frequency band	AECc sensor (SD)	AECc source (SD)	PLI sensor (SD)	PLI source (SD)	AECc sensor (SD)	AECc source (SD)	PLI sensor (SD)	PLI source (SD)
Delta	4.8 (3.8)	2.7 (2.5)	3.6 (2.6)	3.4 (2.4)	4.0 (3.3)	2.9 (2.5)	4.3 (3.3)	3.3 (2.3)
Theta	4.4 (3.1)	3.9 (2.0)	3.7 (3.3)	4.4 (2.7)	6.3 (4.4)	4.4 (3.4)	6.0 (4.5)	4.3 (3.2)
Alpha	6.6 (6.1)	4.7 (3.5)	8.4 (9.3)	4.2 (2.6)	7.2 (6.0)	3.9(2.9)	10.8 (11.1)	3.8 (2.4)
Beta	6.6 (5.6)	4.0 (3.4)	5.1 (5.3)	4.0 (3.2)	6.5 (3.9)	4.7 (2.8)	5.3 (3.9)	3.4 (2.5)

Mean percentage overlap (with standard deviations) of minimum spanning tree (MST) matrices derived from corrected amplitude envelope correlation (AECc) and phase lag index (PLI) measures at both sensor and source level. Overlap is calculated across repeated measurements (6 week interval; N = 42) and across different resting state conditions (resting state vs. semi-resting-state; N = 24) for each frequency band. Higher percentages indicate greater consistency in network topology between measurements. Values are presented as mean percentage ± standard deviation. Sensor and source level analyses are shown separately. *AECc* corrected amplitude envelope correlation, *PLI* phase lag index, *MST* minimum spanning tree, *SD* standard deviation. Frequency bands: delta (0.5–4 Hz), theta (4–8 Hz), alpha (8–13 Hz), and beta (13–20 Hz).

## Discussion

The present study investigated the reliability of quantitative FC, complexity and MST EEG measures. Frequency-dependent reliability was assessed between repeated resting-state EEG measurements over time and between resting-state and semi-resting-state EEG recordings at the same time point, both for sensor-level data and after source reconstruction. The reliability of FC measures varied depending on the measure, frequency band, and application of source reconstruction. Overall, we found moderate to good reliability for FC and complexity measures, where the PE showed highest reliability, but low reliability for MST measures. The results for FC measures were in line with our hypothesis, while PE outperformed our expectation and MST measures performed worse than hypothesized.

In sensor‐level analyses, PLI exhibited somewhat higher ICC values than AECc in the theta and alpha bands, though this pattern varied in other frequency bands and across different states. Additionally, measures in the theta and alpha bands showed higher reliability than in the delta and beta bands. Overall, when comparing ICCs across time and across resting-states, neither was clearly higher than the other. ICCs were slightly higher at sensor level than at source level.

Combining findings of reliability across time and across states, we can categorize measures in terms of their relative state-dependence. The theta and alpha PE showed high reliability across time and states, at both sensor and source level. This indicates PE exhibits high stability across different resting-state conditions, which include both cognitive factors and eye state, essential properties for a potential biomarker. Notably, JPE_INV_ showed lower reliability than PE in all comparisons. It is conceivable that JPE_INV_ is sensitive to non-stationary, metastable brain dynamics^18,55^ which could manifest as lower test-retest reliability. Additionally, it is worth noting that JPE_INV_ is a relatively new FC measure, which means little is known about the influence of factors such as epoch length, signal-to-noise ratio and different parameter settings on reliability. Current evidence does not support the use of JPE_INV_ as a reliable disease marker, though this result can be considered preliminary. Sensor-level theta and alpha PLI showed moderate temporal reliability combined with high reliability across states, implicating PLI as relatively state-independent. All remaining MST measures at source-level showed poor reliability, with low reliability across time and state. It is noteworthy that the task paradigm included eyes open EEG compared to the eyes closed resting-state recordings. Relating to this difference, it has been found that spectral measures in eyes closed resting-state EEG are more stable than in eyes open resting-state EEG^56,57^ and the spatial pattern of oscillatory activity is different between these conditions^4^. Nevertheless, eyes open and eyes closed resting-state EEG differ only marginally in terms of reliability when looking at functional connectivity^15^. Despite this, there is a possibility that some of the EEG measures included in the current study are more sensitive to changes related to eyes open resting-state EEG than others.

There is limited literature to relate our current findings regarding reliability of these EEG measures, but Hommelsen et al. were able to identify an individual neural finger print with high accuracies using a machine learning model based on power spectral density^27^. Interestingly, this was possible both for longitudinal resting-state data and for resting-state versus semi-resting-state data, indicating relative state-independence of the power spectral density when using a machine learning approach. Although not directly comparable to our experiment, previous studies compared reliability of resting-state and task EEG (though not based on a P50-task), reporting heterogeneous results^46,58^.

An additional finding of our study concerns the temporal reliability of EEG measures, which is a fundamental requirement of EEG measures in many applications. At the sensor level, PE in the theta and alpha bands had the highest temporal reliability. PLI in the theta and alpha band together with beta AECc are other, albeit slightly inferior, options. For the same comparison after source reconstruction, PE in the theta and alpha bands was most reliable, with the alpha PLI and beta AECc as a slightly less reliable alternative. From these results, it follows that a general recommendation for longitudinal EEG analyses would be to analyze the theta, alpha and beta bands, favoring PE over more traditional connectivity measures. Although limited evidence exists on the temporal reliability of complexity-based connectivity measures, the finding that alpha PLI demonstrates relatively high temporal reliability in healthy subjects aligns with previous research^8,12,14^. Additionally, the alpha band is known for its robustness and reliability in EEG recordings, and is associated with resting-state neural activity and attentional processes^59^. Regarding reliability of AECc, previous studies explored either magnetoencephalography data^60,61^, simulated data^62^, or looked primarily at reliability between disease states^63^. When looking at the corrected alpha power envelope connectivity, which can be considered equivalent to AECc, our temporal reliability with an ICC of around 0.5 for source-level data is comparable to findings of Rolle and colleagues for source-level EEG data^15^. Concerning the relatively lower reliability of AECc compared to the other measures, we have not quantified the effect of orthogonalization on this result. By comparing reliability of AECc with AEC, this effect could be explored. The finding that the delta band generally showed poor temporal reliability is consistent with its reduced signal-to-noise ratio, which arises from limited delta activity in healthy, awake adults and also from the substantial contamination by artifacts such as eye-blinks and eye movements. Even though MST measures have been found to be promising disease markers^22^, our results indicate that MST EEG measures should be interpreted with caution. The magnitude of MST overlap was similar or slightly higher than previous research in ALS patients^51^, though it remained relatively low. MST using a maximum spanning tree approach is stable and relatively insensitive to small variations in edge weights of the underlying FC matrix^21^. Therefore, both the low MST overlap and relatively low ICC values found for MST measures point towards significant fluctuations in underlying FC matrices between conditions. Interestingly, these fluctuations, especially for PLI, do notseem to result in poor ICC values after averaging the matrix. Together, these findings do not yet support the use of PLI and AECc MST-based EEG measures as possible biomarkers.

A secondary motivation for this study was to compare pure resting‐state data with semi‐resting-state epochs from a P50 gating task. Our findings imply that, for certain measures, there is little penalty in substituting semi‐resting-state for pure resting‐state data. This observation has practical implications, as it suggests that some studies could omit a separate resting-state EEG recording if the semi‐resting-state intervals in a task provide sufficient reliability. On the other hand, for measures that had lower across‐state reliability—such as MST features, FC in delta or beta bands, or certain amplitude‐based metrics—this might not hold true.

In this study, source reconstruction using an LCMV beamforming approach with a template MRI-based Boundary Element Method (BEM) head model did not clearly improve reliability of a wide range of EEG measures. Some considerations are important in this regard. Methods such as beamforming, which attempt to reconstruct source activity by focusing on specific locations within the brain, have been employed with standard head models but still face limitations due to model inaccuracies and assumptions^64^. Alternative source reconstruction approaches incorporate different assumptions about the source activity, leading to different source estimates. For example, Exact Low-Resolution Electromagnetic Tomography (eLORETA) provides smooth solutions for the distribution of neuronal electrical activity^65^. Notably, some studies suggest that Minimum Norm Estimation (MNE) may outperform beamforming techniques in certain contexts, offering better localization accuracy and reliability^15,66,67^. Another comparison between LCMV beamforming, eLORETA and MNE showed comparable between-cohort and within-patient consistency, though LCMV beamforming produced distinctly different FC estimates compared to the other two inverse methods^68^. Incorporating different source reconstruction methods could influence and potentially enhance the reliability of source-level EEG measures.

A strength of this study is that the methodological framework incorporates a wide array of metrics, including PLI, AECc, PE, JPE_INV_ and MST measures, examined at both sensor and source levels, allowing for direct cross‐comparison of reliability. Second, by including both resting‐state and semi‐resting-state data, we could explore whether semi‐resting-state epochs can substitute for purely resting‐state measurements in biomarker‐focused research. Third, our findings of higher reliability in theta and alpha frequencies, especially for PE and PLI, complement earlier reports describing the potential of these bands for identifying stable neural signatures^27^. Finally, the reliability of resting-state EEG across different contexts has been studied sparingly and indirectly. Providing evidence that supports this reliability is of value for the development of clinically relevant biomarkers.

As mentioned previously, the fact that the acrossstate comparison is based on eyes open EEG is a possible physiological confound that complicates interpretation. Another possible limitation of his study is that we did not take into account other factors influencing EEG reliability other than time and state differences, such as sensitivity to noise, coupling strength, spatial leakage, or synchronization transitions^62^. Furthermore, reliability represents only one aspect of what makes an EEG measure valuable and does not address its discriminatory power, despite evidence supporting its ability to discriminate illness effects. In addition, EEG measurement length may have influenced our results, since EEG measures might become more reliable when using longer data segments compared to one minute of data^15^. We made use of a template head model for the beamforming analysis, which is a valid approach for magnetoencephalography. However, this type of evidence does not exist for EEG analyses, and the use of a template head model might influence MST results more than the results for other metrics^69^. We used different sample sizes for the time (n = 42) and state (n = 24) comparisons; ICC values may be under- or overestimated for smaller samples^70^. This can also be observed in the wider confidence intervals regarding the across-state comparisons. Therefore, these results should be interpreted with some caution. An additional limitation is the fact that our cohort consisted of a healthy, relatively young, mostly male population. This limits generalizability of these reliability findings to other clinical populations. Age and illness might influence the reliability of measures like PLI^71^, making the translation to a clinical population uncertain. The effect of sex on our findings is still unknown.

In conclusion, our findings demonstrate that PE in the theta and alpha bands is relatively stable both over time and across different resting‐state contexts, aligning with the requirements of a potential biomarker. To a slightly lesser degree, this also holds true for alpha PLI. For other measures, notably JPE_INV_ and many MST and FC measures in the delta and beta bands, caution is warranted given their lower reliability. Future research is necessary to translate these findings to patient cohorts and to expand the analyses to a more diverse healthy cohort to explore the impact of sex and age.

## Supplementary Information

Below is the link to the electronic supplementary material.

Supplementary Material 1

## Data Availability

The data that support the findings of this study are available on reasonable request from the corresponding author (l.s.dominicus-2@umcutrecht.nl). The data are not publicly available due to their containing information that could compromise the privacy of research participants. All preprocessing and analysis scripts (built with version 1.8.0 of the open-source MNE-Python package; Gramfort et al. 2013) are openly available at https://github.com/yorbenlodema/EEG-Pype.
