# Supplementary material for: Reliability and state-dependency of EEG connectivity, complexity and network characteristics
Source: Sci Rep. 2025 Nov 4;15:38454. doi: 10.1038/s41598-025-23662-z (PMC12586465; doi:10.1038/s41598-025-23662-z)
Supplement: Supplementary file 1 — Supplementary Material 1 [file 41598_2025_23662_MOESM1_ESM.docx]

# Supplements

## S1. EEG preprocessing and analysis

EEG data were preprocessed using functions from version 1.8.0 of the open-source *MNE* Python package [1]. This implementation is openly accessible via: https://github.com/yorbenlodema/EEG-Pype. EEG recordings were first bandpass filtered (0.5-47 Hz), average referenced, and down sampled to 256 Hz to enable fast visual inspection for eye movement and muscle artifacts. Subsequently, the first 15 artifact-free epochs of 4 seconds were selected using manual epoch selection. Channels were interpolated using spherical spline interpolation where necessary (6 channels maximum). We analyzed 60 seconds of data per registration, which is well above the recommended minimum of 32 seconds for test-retest studies of frequency analysis [2]. An epoch length of 4 seconds was chosen because it was previously shown that 12 epochs of 4 seconds have better reliability than 4 epochs of 12 seconds [3, 4]. To avoid event-related signals in the semi-rsEEG, we segmented periods of 4 seconds in the middle of the 10 seconds interval between trials.

For source level analysis, we made use of Linearly Constrained Minimum Variance (LCMV) beamforming [5, 6]. Beamformers are spatially adaptive filters that, in contrast with minimum norm-based approaches, attenuate zero-lag field spread, which otherwise inflates amplitude-based connectivity (AECc) and influences MST measures [5]. Earlier findings support the use of LCMV beamforming with a template MRI which allows for more broad application of source reconstruction [7]. Additionally, prior EEG work shows that LCMV beamforming often yields more reproducible graph-theoretic metrics, especially under reduced electrode density[8]. Furthermore, minimum norm-based approaches have the disadvantage of projecting all data, including artifacts, to the source-space [9], For connectivity analyses in particular, beamformers have demonstrated superior performance over MNE for coherence reconstructions [10]. While there is no single best inverse solution for all applications [11], these factors support the use of LCMV beamforming for the present study. EEG data were first bandpass filtered between 0.5-47 Hz and re-referenced to the average reference. The forward model used was constructed using the FreeSurfer fsaverage template with a three-shell boundary element model consisting of 5120 triangles per layer (brain, skull, and scalp) with MNE default conductivity values. Source locations were defined based on the Desikan-Killiany atlas, using the parcels’ centroids [12], and equivalent current dipoles were used as source models. The beamformer spatial filter was computed using the data covariance matrix and noise covariance matrix (estimated from the diagonal elements of the data covariance), with Tikhonov regularization (parameter=0.05) and unit-noise-gain normalization. Bad (otherwise interpolated) channels were excluded prior to the beamforming computation. The resulting source time courses were projected onto the orientations of maximum power. Since the semi-resting state data was epoched due to the nature of our measurements, we maintained the epoched structure during source reconstruction by calculating the data covariance matrix on the available epochs per participant, allowing for calculation of a spatial filter based on all available epochs. Then, individual epochs were projected through the spatial filter constructed for the participant.

The Phase Lag Index (PLI) and corrected Amplitude Envelope Correlation (AEC) were computed as measures of functional connectivity.

Phase Lag index (PLI)

The PLI is a connectivity measure that indicates the level of coupling between two oscillatory signals (Stam et al., 2007). It can be understood as a measure of the consistency of the phase lags or leads, based on the asymmetry of the distribution of phase differences. Zero-lag phase coupling is discarded and therefore the PLI is less sensitive to volume conduction effects and field spread [13].

The PLI varies from 0 to 1, where 0 indicates no phase synchronization or phase-synchronization with zero-lag, and 1 indicates complete non-zero-lag phase locking.

Amplitude Envelope Correlation (AEC)

The AEC was obtained by estimating the magnitude of the analytic signal, the analytic representation of a real-valued function. A Pearson’s correlation was calculated between the power envelopes of two signals [14].

The corrected version of the AEC (AECc) was used, applying pairwise orthogonalization to the time series before computing the AEC [15, 16]. This means that the signal from one channel is regressed out of another channel, after which the AEC is computed. This process is then repeated in the reverse direction, after which both AEC values are averaged to get the corrected AEC. The computation of orthogonal vectors of the signals combats the effect of spurious synchronization that results from volume conduction/field spread.

Permutation Entropy (PE)

Permutation Entropy (PE), based on the method by Bandt and Pompe [17], was used to quantify the complexity of neural activity. The PE values typically range between 0 and 1, where higher values indicate greater complexity and unpredictability in neural activity and lower PE values suggest more predictable, structured patterns.

When calculating PE, two parameters need to be set: tau and n. Tau specifies the step size, expressed in number of data points, that is used when constructing the permutation patterns. It represents the time delay between consecutive values in the embedding vector. A smaller tau captures faster dynamics in the signal, while a larger tau emphasizes slower oscillatory patterns. The choice of tau is typically based on the sampling rate and the temporal characteristics of interest in the neural data. In our analysis, we set tau = 1/256 s, corresponding to the sampling interval of our recordings.

The parameter n refers to the embedding dimension or the length of patterns used to construct the permutation symbols. It determines how many consecutive data points (with the specified tau interval between them) are used to form each permutation pattern. Higher n values allow for detection of more complex patterns but require longer data segments and increase computational demands. The total number of possible permutation patterns is n!, so the choice of n affects the statistical reliability of the entropy estimation. For our analysis, we selected n = 4, resulting in 24 possible permutation patterns.

Joint permutation entropy (JPE)
The inverted Joint Permutation Entropy (JPE_INV_) extends the concept of PE to multivariate time series [18], examining the relationship between two brain regions. For each region, symbolic representations were generated using PE with a tau of 1/256th of a second, and a probability distribution of combined symbols was constructed to capture the joint dynamics between the two time series. By excluding mirrored and identical symbols to avoid spurious correlations, JPE provided a normalized measure of the shared complexity between brain regions.

The normalization of JPE is achieved by dividing the calculated joint entropy by the theoretical maximum entropy possible for the given embedding dimension. Since we used n = 4, the maximum number of unique joint permutation patterns is (4!)², but after excluding mirrored and identical patterns to prevent artificial correlations, this number is reduced. The resulting normalized JPE ranges from 0 to 1, where 0 indicates perfect synchronization (minimal entropy) and 1 represents complete independence (maximal entropy) between the two time series.

This allowed us to assess the degree of synchronization or independence between different neural areas. Finally, by inverting the JPE (calculated as 1 - JPE), higher values correspond to higher functional connectivity (FC) [19], making the metric more intuitive where higher values indicate stronger connections between brain regions.

Minimum Spanning Tree (MST)

The MST was used to reconstruct a backbone of functional connections, which was subsequently characterized with graph theoretical measures. The MST creates an acyclic sub-network of the brain connecting all nodes, using, typically, the strongest functional connections and reflecting the most fundamental network properties [20, 21]. The use of the MST avoids limitations of other graph theoretical approaches such as sensitivity to connection strengths, arbitrary thresholding, or link density effects [21]. The MST was calculated based on connectivity matrices, which here represented the frequency-specific PLI or AECc. The connectivity matrices consisted of 64 × 64 cells (for 64 EEG sensor-level channels) or 68 x 68 cells (for source-level data) resulting in MSTs with 64 nodes and 63 edges, or 68 nodes and 67 edges, respectively.

## S2. Concatenated corrected amplitude envelope correlation

There are different ways to calculate the AECc. We calculated the AECc using two methods. The first method, as described in the main text, involves calculating the AECc for each epoch individually and then averaging the AECc values across all epochs. We applied the same approach to calculate the other connectivity and complexity measures. In the following section, we will refer to this as the original method. The second approach we used is in line with Rolle et al. [22] where we concatenated the 15 available four-second-long epochs into one continuous data segment of 60 seconds and computed a single AECc value for each participant. We will refer to this as the concatenation method. This approach may lead to a more reliable estimation.

The results are shown in Figures S1. Overall, the AECc results did not improve substantially using this second approach. In some analyses, the first method produced higher ICC values, while in others, the second method performed better.

AECc across time, sensor space

Across time at sensor level, the concatenated AECc and AECc_MST_ showed moderate reliability in the beta band. Compared to the results of the original AECc method, the results were comparable, with slightly higher temporal reliability of AECc and AECc_MST_ with concatenation in the delta and theta band.

AECc across time, source space

When looking into the AECc across time at source level, the theta, alpha and beta band showed moderate reliability for the AECc and AECc_MST_. When comparing this to the original AECc method, the results were comparable or slightly worse than for the original method.

AECc across state, sensor space

Across state at sensor level, the AECc showed moderate to good reliability in the alpha band and beta band. Compared to the original AECc, there appears to be a slight increase in reliability of AECc and AECc_MST_ in all frequency bands. This implies that concatenation prior to AECc calculation might benefit state-independence in sensor-level EEG data.

AECc across state, source space

The AECc across state at source level showed poor reliability across frequency bands. Compared to the original AECc calculation with moderate to good reliability in the alpha and beta band, the reliability of concatenated AECc performed a lot worse. This implicates that for source-level EEG data, non-concatenated AECc is more state-independent.

AECc MST measures

Regarding the MST measures, for all analyses the reliability of the AECc derived measures was generally poor with concatenation prior to AECc. The original AECc method showed slightly higher reliability for some variables, compared to the concatenation method, especially at sensor level.


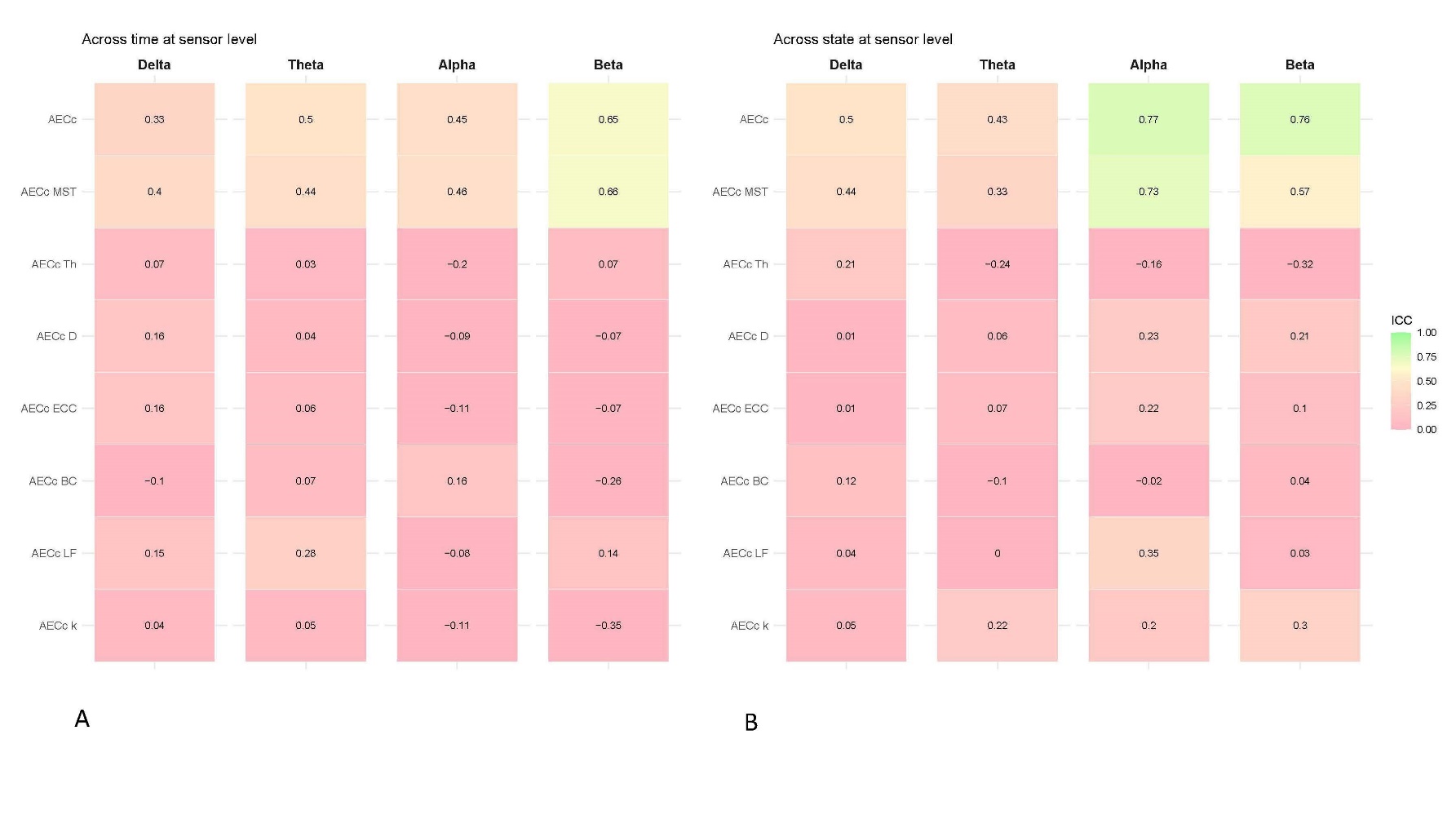

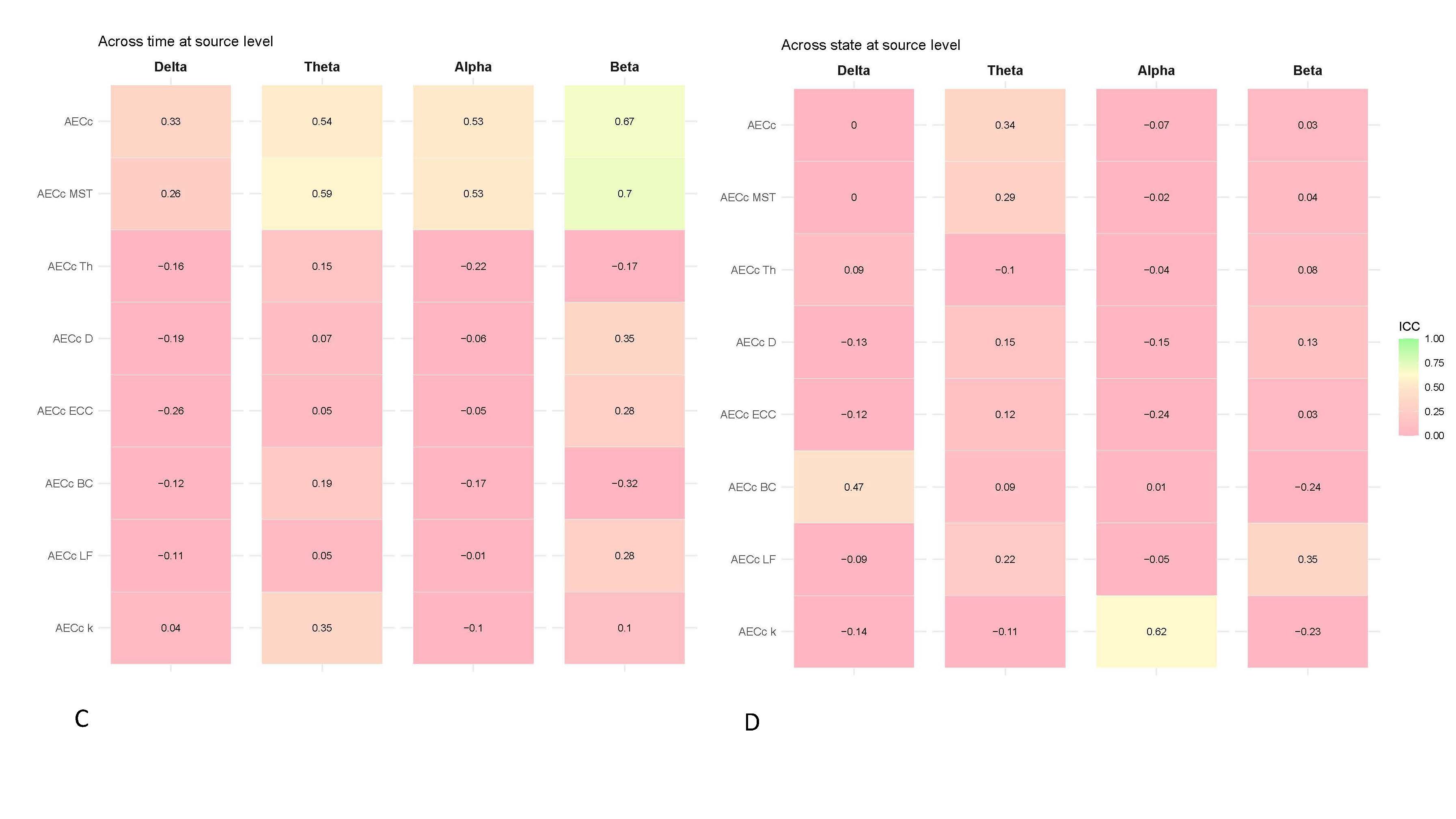


**Figure S1. *Overview of ICC of AECc-based variables, when calculated after concatenating.*** *We concatenated the 15 available four-second-long epochs into one continuous data segment of 60 seconds. We then computed a single AECc value for each participant. A: across time at sensor level, B: across state at sensor level, C: across time at source level, D: across state at source level; MST measures include leaf fraction (LF), maximum degree (k), diameter (D), maximum betweenness centrality (BC), eccentricity (ECC), and tree hierarchy (Th) across different frequency bands (delta: 0.5-4 Hz, theta: 4-8 Hz, alpha: 8-13 Hz, beta: 13-20 Hz. AECc = corrected amplitude envelope correlation.*

## S3. Complete test-retest reliability results

Test-retest reliability was assessed using intraclass correlation coefficients (ICC). The 95% confidence intervals and standard errors for the ICCs were estimated using a non-parametric bootstrap procedure with 1000 resamples. For each resample, subjects were randomly selected with replacement while maintaining their paired test-retest measurements. The ICC was calculated for each bootstrap sample, and the 2.5th and 97.5th percentiles of the bootstrap distribution were used to determine the 95% confidence intervals. The standard error was computed as the difference between the upper and lower confidence intervals divided by 2 × 1.96.

Across time

| **Sensor measure** | **ICC (95% CI)** | **SE** | **Source measure** | **ICC (95% CI)** | **SE** |
| --- | --- | --- | --- | --- | --- |
| Alpha PE | 0.79 (0.68-0.86) | 0.045 | Theta PE | 0.82 (0.71-0.88) | 0.043 |
| Theta PE | 0.79 (0.67-0.87) | 0.050 | Alpha PE | 0.81 (0.68-0.89) | 0.053 |
| Beta PE | 0.75 (0.62-0.84) | 0.054 | Beta AECc (MST) | 0.75 (0.57-0.85) | 0.071 |
| Beta AECc | 0.70 (0.41-0.82) | 0.104 | Beta AECc | 0.74 (0.44-0.85) | 0.104 |
| Beta AECc (MST) | 0.66 (0.43-0.79) | 0.093 | Alpha PLI (MST) | 0.71 (0.59-0.81) | 0.055 |
| Alpha PLI | 0.61 (0.41-0.79) | 0.096 | Alpha PLI | 0.71 (0.55-0.80) | 0.064 |
| Alpha PLI (MST) | 0.60 (0.42-0.77) | 0.090 | Alpha JPE_INV_ | 0.69 (0.52-0.83) | 0.077 |
| Alpha JPE_INV_ | 0.59 (0.39-0.78) | 0.100 | Beta PE | 0.66 (0.52-0.82) | 0.076 |
| Alpha MST k (AECc) | 0.57 (0.38-0.71) | 0.083 | Beta JPE_INV_ | 0.64 (0.39-0.78) | 0.099 |
| Delta PLI (MST) | 0.56 (0.34-0.77) | 0.107 | Theta AECc | 0.60 (0.42-0.75) | 0.084 |
| Theta PLI (MST) | 0.56 (0.29-0.70) | 0.105 | Theta AECc (MST) | 0.60 (0.41-0.75) | 0.086 |
| Theta MST LF (PLI) | 0.51 (0.28-0.70) | 0.106 | Theta PLI (MST) | 0.57 (0.21-0.72) | 0.129 |
| Theta PLI | 0.51 (0.17-0.70) | 0.135 | Beta PLI | 0.57 (0.24-0.74) | 0.125 |
| Theta JPE_INV_ | 0.48 (0.35-0.69) | 0.085 | Beta PLI (MST) | 0.55 (0.32-0.70) | 0.096 |
| Alpha AECc (MST) | 0.46 (0.27-0.65) | 0.098 | Theta JPE_INV_ | 0.54 (0.29-0.68) | 0.099 |
| Alpha AECc | 0.45 (0.28-0.70) | 0.107 | Alpha AECc (MST) | 0.54 (0.36-0.68) | 0.082 |
| Alpha MST k (PLI) | 0.45 (0.23-0.60) | 0.096 | Alpha AECc | 0.52 (0.31-0.67) | 0.090 |
| Beta JPE_INV_ | 0.44 (0.19-0.65) | 0.117 | Alpha MST LF (AECc) | 0.51 (0.35-0.63) | 0.070 |
| Delta MST Th (PLI) | 0.43 (0.17-0.61) | 0.111 | Theta PLI | 0.44 (0.09-0.63) | 0.137 |
| Delta MST LF (PLI) | 0.40 (0.19-0.55) | 0.093 | Delta PE | 0.43 (0.23-0.63) | 0.101 |
| Theta MST k (PLI) | 0.39 (0.12-0.59) | 0.121 | Alpha MST ECC (AECc) | 0.43 (0.02-0.68) | 0.166 |
| Beta MST LF (PLI) | 0.39 (0.09-0.56) | 0.120 | Alpha MST D (AECc) | 0.41 (0.04-0.67) | 0.163 |
| Theta MST Th (AECc) | 0.38 (0.15-0.60) | 0.116 | Beta MST LF (PLI) | 0.41 (0.21-0.57) | 0.093 |
| Beta PLI (MST) | 0.37 (0.10-0.56) | 0.117 | Alpha MST LF (PLI) | 0.39 (0.19-0.55) | 0.092 |
| Theta AECc | 0.37 (0.09-0.65) | 0.143 | Beta MST k (AECc) | 0.37 (0.13-0.61) | 0.120 |
| Alpha MST LF (AECc) | 0.37 (0.08-0.57) | 0.124 | Delta PLI (MST) | 0.37 (0.17-0.63) | 0.119 |
| Delta MST ECC (AECc) | 0.36 (0.09-0.57) | 0.123 | Alpha MST Th (AECc) | 0.36 (0.18-0.56) | 0.095 |
| Delta MST D (AECc) | 0.34 (0.06-0.54) | 0.123 | Delta MST k (AECc) | 0.34 (0.09-0.53) | 0.111 |
| Theta MST LF (AECc) | 0.34 (0.13-0.57) | 0.114 | Delta AECc | 0.34 (0.03-0.51) | 0.124 |
| Beta MST LF (AECc) | 0.34 (0.13-0.50) | 0.094 | Beta MST LF (AECc) | 0.31 (0.07-0.49) | 0.105 |
| Theta MST D (PLI) | 0.32 (0.12-0.54) | 0.107 | Beta MST k (PLI) | 0.30 (0.12-0.49) | 0.096 |
| Alpha MST BC (PLI) | 0.32 (-0.07-0.54) | 0.157 | Theta MST ECC (AECc) | 0.29 (0.11-0.45) | 0.087 |
| Delta PLI | 0.32 (0.04-0.60) | 0.145 | Alpha MST Th (PLI) | 0.28 (0.11-0.43) | 0.081 |
| Alpha MST ECC (AECc) | 0.32 (0.12-0.51) | 0.100 | Alpha MST k (PLI) | 0.28 (0.06-0.45) | 0.099 |
| Theta AECc (MST) | 0.31 (0.06-0.62) | 0.145 | Theta MST D (AECc) | 0.27 (0.09-0.46) | 0.093 |
| Beta PLI | 0.31 (0.00-0.55) | 0.139 | Alpha MST k (AECc) | 0.26 (0.01-0.47) | 0.119 |
| Alpha MST LF (PLI) | 0.31 (0.11-0.49) | 0.098 | Alpha MST BC (AECc) | 0.26 (0.04-0.45) | 0.105 |
| Delta MST k (PLI) | 0.30 (0.10-0.45) | 0.090 | Delta PLI | 0.25 (0.03-0.55) | 0.133 |
| Delta MST Th (AECc) | 0.29 (0.00-0.52) | 0.133 | Theta MST LF (AECc) | 0.24 (0.03-0.43) | 0.103 |
| Theta MST ECC (PLI) | 0.29 (0.08-0.48) | 0.102 | Delta JPEINV | 0.23 (0.03-0.44) | 0.103 |
| Alpha MST BC (AECc) | 0.28 (0.04-0.46) | 0.107 | Delta AECc (MST) | 0.23 (0.03-0.44) | 0.106 |
| Theta MST ECC (AECc) | 0.28 (0.07-0.48) | 0.104 | Beta MST Th (PLI) | 0.20 (-0.03-0.43) | 0.118 |
| Beta MST ECC (AECc) | 0.28 (0.04-0.49) | 0.115 | Alpha MST ECC (PLI) | 0.20 (0.02-0.33) | 0.079 |
| Theta MST D (AECc) | 0.24 (0.00-0.46) | 0.118 | Alpha MST D (PLI) | 0.19 (-0.02-0.35) | 0.095 |
| Alpha MST D (AECc) | 0.24 (-0.02-0.45) | 0.119 | Beta MST D (AECc) | 0.18 (-0.05-0.39) | 0.113 |
| Delta MST LF (AECc) | 0.23 (-0.11-0.48) | 0.150 | Theta MST Th (PLI) | 0.17 (-0.04-0.34) | 0.098 |
| Beta MST D (AECc) | 0.22 (-0.02-0.45) | 0.121 | Theta MST k (AECc) | 0.15 (-0.14-0.39) | 0.133 |
| Alpha MST Th (AECc) | 0.21 (-0.10-0.46) | 0.142 | Beta MST ECC (AECc) | 0.15 (-0.08-0.36) | 0.114 |
| Delta MST BC (AECc) | 0.21 (0.02-0.41) | 0.100 | Beta MST Th (AECc) | 0.15 (-0.04-0.34) | 0.096 |
| Beta MST Th (AECc) | 0.21 (-0.04-0.45) | 0.124 | Delta MST LF (PLI) | 0.12 (-0.15-0.32) | 0.119 |
| Beta MST k (PLI) | 0.20 (-0.14-0.44) | 0.146 | Delta MST k (PLI) | 0.12 (-0.15-0.38) | 0.136 |
| Alpha MST D (PLI) | 0.20 (-0.11-0.43) | 0.138 | Delta MST LF (AECc) | 0.11 (-0.14-0.29) | 0.111 |
| Alpha MST ECC (PLI) | 0.19 (-0.08-0.40) | 0.123 | Theta MST BC (AECc) | 0.09 (-0.15-0.30) | 0.116 |
| Theta MST Th (PLI) | 0.18 (-0.03-0.38) | 0.106 | Theta MST ECC (PLI) | 0.09 (-0.14-0.30) | 0.113 |
| Delta MST BC (PLI) | 0.18 (-0.12-0.41) | 0.134 | Theta MST D (PLI) | 0.07 (-0.18-0.28) | 0.118 |
| Beta MST Th (PLI) | 0.16 (-0.06-0.35) | 0.105 | Beta MST D (PLI) | 0.06 (-0.18-0.26) | 0.111 |
| Delta PE | 0.11 (-0.12-0.38) | 0.127 | Theta MST k (PLI) | 0.05 (-0.20-0.30) | 0.126 |
| Delta AECc (MST) | 0.09 (-0.06-0.36) | 0.108 | Theta MST LF (PLI) | 0.05 (-0.25-0.33) | 0.148 |
| Delta MST ECC (PLI) | 0.07 (-0.22-0.31) | 0.135 | Beta MST BC (PLI) | 0.05 (-0.25-0.34) | 0.151 |
| Delta MST k (AECc) | 0.07 (-0.12-0.28) | 0.102 | Beta MST ECC (PLI) | 0.04 (-0.19-0.24) | 0.110 |
| Beta MST ECC (PLI) | 0.05 (-0.19-0.25) | 0.112 | Delta MST BC (PLI) | 0.03 (-0.27-0.26) | 0.136 |
| Alpha MST Th (PLI) | 0.05 (-0.23-0.23) | 0.117 | Delta MST D (PLI) | 0.03 (-0.21-0.30) | 0.132 |
| Delta MST D (PLI) | 0.05 (-0.26-0.27) | 0.135 | Delta MST ECC (PLI) | 0.01 (-0.25-0.29) | 0.137 |
| Delta AECc | 0.04 (-0.17-0.40) | 0.147 | Delta MST BC (AECc) | 0.00 (-0.23-0.22) | 0.115 |
| Theta MST k (AECc) | 0.03 (-0.16-0.20) | 0.093 | Theta MST Th (AECc) | -0.01 (-0.27-0.25) | 0.132 |
| Theta MST BC (PLI) | 0.03 (-0.18-0.22) | 0.101 | Beta MST BC (AECc) | -0.01 (-0.21-0.23) | 0.113 |
| Beta MST D (PLI) | 0.01 (-0.22-0.22) | 0.111 | Alpha MST BC (PLI) | -0.09 (-0.27-0.16) | 0.111 |
| Delta JPE_INV_ | -0.04 (-0.22-0.20) | 0.108 | Delta MST Th (AECc) | -0.10 (-0.33-0.09) | 0.108 |
| Beta MST BC (AECc) | -0.07 (-0.31-0.15) | 0.118 | Delta MST Th (PLI) | -0.11 (-0.37-0.13) | 0.128 |
| Theta MST BC (AECc) | -0.08 (-0.33-0.15) | 0.124 | Theta MST BC (PLI) | -0.16 (-0.39-0.06) | 0.114 |
| Beta MST k (AECc) | -0.12 (-0.34-0.11) | 0.115 | Delta MST D (AECc) | -0.17 (-0.37-0.07) | 0.112 |
| Beta MST BC (PLI) | -0.30 (-0.59--0.00) | 0.150 | Delta MST ECC (AECc) | -0.17 (-0.39-0.07) | 0.116 |

Across state

| **Sensor measure** | **ICC (95% CI)** | **SE** | **Source measure** | **ICC (95% CI)** | **SE** |
| --- | --- | --- | --- | --- | --- |
| Theta PLI | 0.94 (0.35-0.97) | 0.159 | Alpha PE | 0.83 (0.70-0.92) | 0.056 |
| Theta PLI (MST) | 0.94 (0.51-0.97) | 0.117 | Theta PLI (MST) | 0.80 (0.30-0.90) | 0.152 |
| Alpha PE | 0.88 (0.77-0.93) | 0.041 | Theta PE | 0.79 (0.61-0.90) | 0.073 |
| Theta PE | 0.87 (0.75-0.92) | 0.043 | Alpha AECc | 0.79 (0.51-0.89) | 0.097 |
| Alpha PLI | 0.80 (0.65-0.90) | 0.064 | Alpha AECc (MST) | 0.75 (0.54-0.85) | 0.078 |
| Theta MST LF (PLI) | 0.79 (0.53-0.88) | 0.089 | Theta PLI | 0.69 (-0.06-0.90) | 0.243 |
| Alpha PLI (MST) | 0.79 (0.63-0.90) | 0.069 | Alpha PLI (MST) | 0.67 (0.45-0.86) | 0.104 |
| Theta MST k (PLI) | 0.76 (0.51-0.81) | 0.078 | Delta PE | 0.66 (0.48-0.81) | 0.084 |
| Alpha MST LF (AECc) | 0.73 (0.38-0.84) | 0.116 | Alpha PLI | 0.63 (0.42-0.86) | 0.112 |
| Alpha AECc | 0.69 (0.41-0.84) | 0.111 | Beta PE | 0.60 (0.33-0.78) | 0.117 |
| Alpha MST k (PLI) | 0.68 (0.46-0.87) | 0.105 | Beta PLI | 0.56 (0.22-0.76) | 0.139 |
| Delta MST LF (AECc) | 0.67 (0.40-0.86) | 0.116 | Beta AECc | 0.55 (0.44-0.79) | 0.089 |
| Alpha MST LF (PLI) | 0.66 (0.40-0.83) | 0.111 | Beta AECc (MST) | 0.55 (0.42-0.66) | 0.063 |
| Beta PE | 0.64 (0.51-0.74) | 0.058 | Beta PLI (MST) | 0.49 (0.24-0.64) | 0.101 |
| Theta MST D (PLI) | 0.63 (0.07-0.79) | 0.186 | Delta AECc | 0.43 (0.05-0.72) | 0.173 |
| Theta MST LF (AECc) | 0.63 (0.31-0.80) | 0.123 | Delta AECc (MST) | 0.42 (0.08-0.62) | 0.137 |
| Alpha AECc (MST) | 0.61 (0.25-0.82) | 0.145 | Delta MST D (AECc) | 0.34 (0.04-0.56) | 0.134 |
| Delta PLI (MST) | 0.61 (0.25-0.75) | 0.127 | Theta MST Th (PLI) | 0.34 (-0.04-0.64) | 0.174 |
| Theta MST ECC (PLI) | 0.60 (0.02-0.77) | 0.193 | Theta MST D (AECc) | 0.33 (0.07-0.52) | 0.113 |
| Alpha MST ECC (PLI) | 0.57 (0.20-0.81) | 0.157 | Alpha MST LF (PLI) | 0.32 (0.10-0.51) | 0.104 |
| Delta PLI | 0.55 (0.14-0.62) | 0.123 | Theta MST ECC (PLI) | 0.31 (-0.20-0.61) | 0.205 |
| Beta MST Th (AECc) | 0.54 (0.22-0.79) | 0.146 | Theta MST D (PLI) | 0.31 (-0.18-0.58) | 0.193 |
| Delta MST ECC (AECc) | 0.53 (0.18-0.77) | 0.151 | Beta MST ECC (PLI) | 0.31 (0.07-0.52) | 0.116 |
| Alpha MST D (PLI) | 0.53 (0.09-0.81) | 0.184 | Delta MST ECC (AECc) | 0.30 (0.04-0.54) | 0.128 |
| Delta MST D (AECc) | 0.53 (0.22-0.77) | 0.140 | Theta MST ECC (AECc) | 0.30 (0.05-0.47) | 0.108 |
| Beta MST LF (AECc) | 0.52 (0.10-0.81) | 0.180 | Delta PLI | 0.30 (0.09-0.47) | 0.099 |
| Beta PLI | 0.52 (0.25-0.73) | 0.122 | Delta MST LF (AECc) | 0.28 (-0.08-0.55) | 0.159 |
| Delta AECc (MST) | 0.50 (0.27-0.71) | 0.113 | Theta MST LF (PLI) | 0.28 (0.00-0.48) | 0.124 |
| Beta PLI (MST) | 0.49 (0.16-0.73) | 0.147 | Beta MST D (PLI) | 0.27 (0.02-0.49) | 0.121 |
| Beta AECc | 0.48 (0.39-0.56) | 0.042 | Theta MST BC (AECc) | 0.26 (-0.12-0.55) | 0.169 |
| Theta MST k (AECc) | 0.47 (0.13-0.76) | 0.160 | Alpha MST Th (AECc) | 0.26 (0.03-0.45) | 0.107 |
| Delta MST Th (AECc) | 0.46 (0.17-0.66) | 0.125 | Alpha MST Th (PLI) | 0.25 (0.02-0.46) | 0.114 |
| Delta MST D (PLI) | 0.46 (0.15-0.68) | 0.137 | Alpha MST ECC (PLI) | 0.25 (-0.11-0.52) | 0.159 |
| Delta MST ECC (PLI) | 0.42 (0.01-0.65) | 0.164 | Alpha MST D (PLI) | 0.24 (-0.13-0.53) | 0.169 |
| Alpha MST Th (AECc) | 0.41 (-0.08-0.66) | 0.189 | Delta MST Th (AECc) | 0.23 (-0.16-0.60) | 0.194 |
| Theta MST BC (PLI) | 0.41 (0.06-0.58) | 0.135 | Delta MST LF (PLI) | 0.23 (-0.17-0.56) | 0.185 |
| Theta MST Th (AECc) | 0.41 (0.09-0.61) | 0.132 | Beta MST LF (AECc) | 0.22 (-0.01-0.46) | 0.118 |
| Beta MST LF (PLI) | 0.40 (0.06-0.59) | 0.137 | Theta AECc | 0.22 (0.13-0.32) | 0.048 |
| Delta MST LF (PLI) | 0.40 (0.12-0.61) | 0.124 | Delta PLI (MST) | 0.22 (-0.01-0.41) | 0.108 |
| Delta MST k (AECc) | 0.39 (0.18-0.71) | 0.137 | Theta MST LF (AECc) | 0.21 (-0.07-0.54) | 0.154 |
| Theta MST Th (PLI) | 0.36 (0.01-0.61) | 0.152 | Alpha MST LF (AECc) | 0.21 (0.02-0.44) | 0.108 |
| Beta MST ECC (AECc) | 0.36 (0.14-0.57) | 0.110 | Theta MST BC (PLI) | 0.20 (-0.34-0.53) | 0.223 |
| Theta AECc | 0.36 (0.23-0.64) | 0.105 | Delta MST D (PLI) | 0.19 (-0.10-0.44) | 0.138 |
| Alpha MST k (AECc) | 0.35 (0.11-0.59) | 0.123 | Alpha MST k (PLI) | 0.17 (-0.11-0.49) | 0.152 |
| Beta MST k (AECc) | 0.35 (-0.01-0.72) | 0.184 | Delta MST k (AECc) | 0.17 (-0.21-0.45) | 0.167 |
| Delta AECc | 0.34 (0.11-0.60) | 0.124 | Delta MST ECC (PLI) | 0.16 (-0.14-0.45) | 0.148 |
| Alpha MST BC (PLI) | 0.33 (0.01-0.54) | 0.135 | Beta MST BC (AECc) | 0.15 (-0.29-0.48) | 0.196 |
| Alpha MST ECC (AECc) | 0.33 (0.11-0.51) | 0.102 | Theta MST k (AECc) | 0.13 (-0.17-0.58) | 0.194 |
| Beta AECc (MST) | 0.32 (0.17-0.42) | 0.065 | Beta MST BC (PLI) | 0.11 (-0.18-0.42) | 0.153 |
| Beta MST D (AECc) | 0.30 (0.05-0.52) | 0.118 | Theta AECc (MST) | 0.10 (0.01-0.16) | 0.038 |
| Beta MST BC (AECc) | 0.29 (0.01-0.53) | 0.133 | Delta MST Th (PLI) | 0.10 (-0.44-0.48) | 0.234 |
| Alpha JPE_INV_ | 0.28 (0.11-0.36) | 0.064 | Delta MST k (PLI) | 0.10 (-0.16-0.40) | 0.142 |
| Alpha MST D (AECc) | 0.28 (0.03-0.44) | 0.105 | Alpha JPE_INV_ | 0.08 (0.04-0.11) | 0.019 |
| Delta MST k (PLI) | 0.27 (-0.20-0.52) | 0.184 | Theta MST k (PLI) | 0.06 (-0.20-0.33) | 0.137 |
| Delta PE | 0.27 (0.06-0.54) | 0.124 | Beta MST ECC (AECc) | 0.06 (-0.30-0.53) | 0.210 |
| Delta MST Th (PLI) | 0.25 (-0.06-0.47) | 0.134 | Beta MST D (AECc) | 0.05 (-0.26-0.47) | 0.186 |
| Alpha MST Th (PLI) | 0.23 (-0.11-0.41) | 0.133 | Beta MST LF (PLI) | 0.04 (-0.18-0.41) | 0.149 |
| Beta MST k (PLI) | 0.21 (-0.10-0.50) | 0.152 | Theta JPE_INV_ | 0.04 (0.00-0.07) | 0.018 |
| Theta MST D (AECc) | 0.21 (-0.19-0.48) | 0.171 | Theta MST Th (AECc) | 0.03 (-0.30-0.40) | 0.176 |
| Theta MST ECC (AECc) | 0.21 (-0.17-0.48) | 0.165 | Alpha MST BC (PLI) | 0.02 (-0.41-0.45) | 0.220 |
| Beta MST Th (PLI) | 0.20 (-0.08-0.39) | 0.119 | Delta MST BC (AECc) | 0.02 (-0.33-0.35) | 0.173 |
| Theta AECc (MST) | 0.18 (0.06-0.39) | 0.083 | Beta JPE_INV_ | 0.01 (0.00-0.01) | 0.002 |
| Delta MST BC (AECc) | 0.15 (-0.15-0.42) | 0.146 | Beta MST k (PLI) | 0.00 (-0.23-0.36) | 0.151 |
| Theta JPE_INV_ | 0.11 (0.00-0.18) | 0.044 | Delta JPE_INV_ | 0.00 (0.00-0.00) | 0.001 |
| Alpha MST BC (AECc) | 0.09 (-0.17-0.34) | 0.130 | Alpha MST k (AECc) | -0.03 (-0.25-0.30) | 0.142 |
| Delta MST BC (PLI) | 0.08 (-0.35-0.39) | 0.189 | Beta MST k (AECc) | -0.08 (-0.38-0.30) | 0.175 |
| Beta JPE_INV_ | 0.01 (0.00-0.02) | 0.004 | Beta MST Th (PLI) | -0.12 (-0.37-0.34) | 0.181 |
| Delta JPEINV | 0.00 (0.00-0.00) | 0.000 | Beta MST Th (AECc) | -0.15 (-0.50-0.25) | 0.191 |
| Beta MST BC (PLI) | -0.04 (-0.33-0.37) | 0.177 | Alpha MST BC (AECc) | -0.16 (-0.55-0.20) | 0.189 |
| Beta MST ECC (PLI) | -0.05 (-0.36-0.25) | 0.154 | Alpha MST D (AECc) | -0.17 (-0.43-0.18) | 0.155 |
| Beta MST D (PLI) | -0.11 (-0.44-0.19) | 0.162 | Alpha MST ECC (AECc) | -0.20 (-0.47-0.09) | 0.142 |
| Theta MST BC (AECc) | -0.24 (-0.50-0.07) | 0.145 | Delta MST BC (PLI) | -0.24 (-0.47-0.09) | 0.142 |

## References

[1] A. Gramfort, “MEG and EEG data analysis with MNE-Python,” *Front Neurosci*, vol. 7, 2013, doi: 10.3389/fnins.2013.00267.

[2] W. Duan, X. Chen, Y. J. Wang, W. Zhao, H. Yuan, and X. Lei, “Reproducibility of power spectrum, functional connectivity and network construction in resting-state EEG,” *J Neurosci Methods*, vol. 348, 2021, doi: 10.1016/j.jneumeth.2020.108985.

[3] M. Hardmeier, F. Hatz, H. Bousleiman, C. Schindler, C. J. Stam, and P. Fuhr, “Reproducibility of functional connectivity and graph measures based on the phase lag index (PLI) and weighted phase lag index (wPLI) derived from high resolution EEG,” *PLoS One*, vol. 9, no. 10, 2014, doi: 10.1371/journal.pone.0108648.

[4] M. Fraschini, M. Demuru, A. Crobe, F. Marrosu, C. J. Stam, and A. Hillebrand, “The effect of epoch length on estimated EEG functional connectivity and brain network organisation,” *J Neural Eng*, vol. 13, no. 3, p. 036015, Jun. 2016, doi: 10.1088/1741-2560/13/3/036015.

[5] B. D. Van Veen, W. Van Drongelen, M. Yuchtman, and A. Suzuki, “Localization of brain electrical activity via linearly constrained minimum variance spatial filtering,” *IEEE Trans Biomed Eng*, vol. 44, no. 9, 1997, doi: 10.1109/10.623056.

[6] A. Hillebrand and G. R. Barnes, “Beamformer Analysis of MEG Data,” 2005. doi: 10.1016/S0074-7742(05)68006-3.

[7] L. Douw, D. Nieboer, C. J. Stam, P. Tewarie, and A. Hillebrand, “Consistency of magnetoencephalographic functional connectivity and network reconstruction using a template versus native M <scp>RI</scp> for co‐registration,” *Hum Brain Mapp*, vol. 39, no. 1, pp. 104–119, Jan. 2018, doi: 10.1002/hbm.23827.

[8] C. Hatlestad-Hall *et al.*, “Reliable evaluation of functional connectivity and graph theory measures in source-level EEG: How many electrodes are enough?,” *Clinical Neurophysiology*, vol. 150, 2023, doi: 10.1016/j.clinph.2023.03.002.

[9] S. Baillet, J. C. Mosher, and R. M. Leahy, “Electromagnetic brain mapping,” *IEEE Signal Process Mag*, vol. 18, no. 6, pp. 14–30, 2001, doi: 10.1109/79.962275.

[10] A. S. Hincapié *et al.*, “The impact of MEG source reconstruction method on source-space connectivity estimation: A comparison between minimum-norm solution and beamforming,” *Neuroimage*, vol. 156, 2017, doi: 10.1016/j.neuroimage.2017.04.038.

[11] L. Tait, A. Özkan, M. J. Szul, and J. Zhang, “A systematic evaluation of source reconstruction of resting MEG of the human brain with a new high-resolution atlas: Performance, precision, and parcellation,” *Hum Brain Mapp*, vol. 42, no. 14, 2021, doi: 10.1002/hbm.25578.

[12] A. Hillebrand *et al.*, “Direction of information flow in large-scale resting-state networks is frequency-dependent,” *Proc Natl Acad Sci U S A*, 2016, doi: 10.1073/pnas.1515657113.

[13] M. Lai, M. Demuru, A. Hillebrand, and M. Fraschini, “A comparison between scalp- and source-reconstructed EEG networks,” *Sci Rep*, vol. 8, no. 1, 2018, doi: 10.1038/s41598-018-30869-w.

[14] A. Bruns, R. Eckhorn, H. Jokeit, and A. Ebner, “Amplitude envelope correlation detects coupling among incoherent brain signals,” *Neuroreport*, vol. 11, no. 7, 2000, doi: 10.1097/00001756-200005150-00029.

[15] J. F. Hipp, D. J. Hawellek, M. Corbetta, M. Siegel, and A. K. Engel, “Large-scale cortical correlation structure of spontaneous oscillatory activity,” *Nat Neurosci*, 2012, doi: 10.1038/nn.3101.

[16] M. Fraschini, S. M. Pani, L. Didaci, and G. L. Marcialis, “Robustness of functional connectivity metrics for EEG-based personal identification over task-induced intra-class and inter-class variations,” *Pattern Recognit Lett*, 2019, doi: 10.1016/j.patrec.2019.03.025.

[17] C. Bandt and B. Pompe, “Permutation Entropy: A Natural Complexity Measure for Time Series,” *Phys Rev Lett*, vol. 88, no. 17, 2002, doi: 10.1103/PhysRevLett.88.174102.

[18] Y. Yin, P. Shang, A. C. Ahn, and C. K. Peng, “Multiscale joint permutation entropy for complex time series,” *Physica A: Statistical Mechanics and its Applications*, vol. 515, 2019, doi: 10.1016/j.physa.2018.09.179.

[19] E. P. Scheijbeler, A. M. van Nifterick, C. J. Stam, A. Hillebrand, A. A. Gouw, and W. de Haan, “Network-level permutation entropy of resting-state MEG recordings: A novel biomarker for early-stage Alzheimer’s disease?,” *Network Neuroscience*, vol. 6, no. 2, 2022, doi: 10.1162/netn_a_00224.

[20] J. B. Kruskal, “On the Shortest Spanning Subtree of a Graph and the Traveling Salesman Problem (1956),” in *Ideas That Created the Future*, 2021. doi: 10.7551/mitpress/12274.003.0019.

[21] P. Tewarie, E. van Dellen, A. Hillebrand, and C. J. Stam, “The minimum spanning tree: An unbiased method for brain network analysis,” *Neuroimage*, 2015, doi: 10.1016/j.neuroimage.2014.10.015.

[22] C. E. Rolle *et al.*, “Functional connectivity using high density EEG shows competitive reliability and agreement across test/retest sessions,” *J Neurosci Methods*, vol. 367, 2022, doi: 10.1016/j.jneumeth.2021.109424.
